# Differentiating Kawasaki disease from urinary tract infection in febrile children with pyuria and C-reactive protein elevation

**DOI:** 10.1186/s13052-018-0585-7

**Published:** 2018-11-20

**Authors:** Seung Beom Han, Soo-Young Lee

**Affiliations:** 1 Department of Pediatrics, College of Medicine, The Catholic University of Korea, Seoul, Republic of Korea; 2 Department of Pediatrics, St. Paul’s Hospital, College of Medicine, The Catholic University of Korea, 180 Wangsan-ro, Dongdaemun-gu, Seoul, 02559 Republic of Korea

**Keywords:** Kawasaki disease, Urinary tract infection, Pyuria, C-reactive protein

## Abstract

**Background:**

Kawasaki disease (KD) is sometimes confused with urinary tract infection (UTI) because both can present with pyuria and C-reactive protein (CRP) elevation. The present study investigated the clinical and laboratory findings that can differentiate KD from UTI in febrile children with pyuria and CRP elevation.

**Methods:**

Medical records were retrospectively reviewed for children with KD and those with UTI. The clinical and laboratory findings between the KD with pyuria group (*n* = 48) and the UTI group (*n* = 118) were compared.

**Results:**

The KD with pyuria group had older age (*P* < 0.001) and longer duration of fever (*P* < 0.001) than the UTI group. In blood tests, both groups showed increased CRP level, but the value of CRP was higher in the KD with pyuria group than in the UTI group (*P* < 0.001). The KD with pyuria group also showed higher values for liver enzymes than the UTI group (*P* < 0.001); > 70.0% of children in the KD with pyuria group, but < 20.0% of children in the UTI group possessed elevated liver enzymes (*P* < 0.001). On urinalysis, 40.7% of the UTI group had a positive nitrite test, but 0.0% of the KD with pyuria group had a positive nitrite test (*P* < 0.001).

**Conclusions:**

Elevated liver enzymes are more specific to KD than to UTI, whereas a positive nitrite test is more specific to UTI than to KD. Our findings can be used as diagnostic clues to differentiate KD from UTI in febrile children with pyuria and CRP elevation.

## Background

Kawasaki disease (KD) is an acute febrile illness that predominantly occurs in children under 5 years of age [1]. It is characterized by prolonged fever and a collection of clinical features (conjunctivitis, oropharyngeal inflammation, skin rash, changes of the extremities, and cervical lymphadenopathy), which comprises the current diagnostic criteria [1, 2]. Coronary artery lesions (CALs) develop in 15–25% of untreated children and can lead to long-term cardiovascular complications [3, 4]. Therefore, early diagnosis and timely initiation of treatment with intravenous immunoglobulin (IVIG) are the greatest priorities in the management of KD [1, 4]. However, diagnosis of KD is often challenging because the diagnostic criteria for KD do not identify all children with the disease [5]. To support the diagnosis of incomplete KD (also sometimes referred to atypical KD) in a child whose clinical presentation suggests KD but whose clinical features do not meet the criteria, compatible echocardiographic or laboratory findings are used [6, 7].

Pyuria is seen in 33 to 63% of children at the initial stage of KD and, therefore, is a clue in the evaluation of a patient suspected with incomplete KD [8, 9]. However, at the same time, it is a pitfall in the misdiagnosis of KD as urinary tract infection (UTI), especially in a febrile child who does not show the typical features of KD but does have pyuria and C-reactive protein (CRP) elevation [10–13]. In the present study, we aimed to determine the clinical and laboratory findings that can differentiate KD from UTI in febrile children with pyuria and CRP elevation.

## Methods

### Study population

Medical records of 140 children who were diagnosed with KD at the Department of Pediatrics, St. Paul’s Hospital, Seoul, Republic of Korea, between January 2015 and December 2016 were retrospectively reviewed. The diagnosis of KD was based on the American Heart Association criteria [1]. Typical KD was diagnosed by the presence of fever and ≥ 4 of 5 principal clinical features. Incomplete KD was diagnosed by the presence of fever and < 4 of 5 principal clinical features plus compatible echocardiographic or laboratory findings: anemia, increased white blood cell (WBC) count, thrombocytosis, hypoalbuminemia, elevated liver enzymes, or pyuria. All children with KD received treatment with IVIG (2 g/kg/dose) and aspirin (30–50 mg/kg/day) and underwent echocardiography to assess coronary artery status during hospitalization. Among the 140 children with KD, 48 cases (34.3%) who presented with pyuria (≥10 WBCs/high-power field) were allocated to the KD with pyuria group.

As a control group (UTI group), 118 children who were diagnosed with UTI at the same hospital between January 2016 and December 2016 were selected. The diagnosis of UTI was based on identification of both pyuria and a single urinary pathogen with > 10^5^ colony forming units/mL in a urine culture [14, 15]. Urine samples were collected from non-toilet-trained children by applying a sterile urine bag over the perineum after skin disinfection, and clean, voided, midstream urine was collected from toilet-trained children [16].

### Data collection

Clinical findings of age, sex, duration of fever at diagnosis, length of hospital stay, and antibiotic usage during the hospitalization were documented for all children. The laboratory findings on the day of admission were recorded and comprised hemoglobin, WBC count, platelet count, CRP, sodium, albumin, aspartate transaminase (AST), and alanine transaminase (ALT) from the blood and nitrite test and the presence of pyuria, hematuria (> 5 red blood cells/high-power field), and proteinuria (> 30 mg/dL) from the urine [9].

For children with KD and pyuria, coronary artery status and response to IVIG therapy were separately examined. Coronary artery dilation was defined as internal diameter up to 1.5 times that of the normal upper limit, and coronary artery aneurysm was defined as internal diameter greater than 1.5 times that of the normal upper limit [3]. Unresponsiveness to IVIG therapy was defined as fever at least 36 h after the first dose of IVIG [1].

### Statistical analysis

A χ2 test was used to compare categorical variables, while continuous variables were compared using the Mann-Whitney *U* test. All tests were 2-tailed, and a *P* value < 0.05 was considered statistically significant. Statistical analyses were performed using SPSS version 18.0 (IBM Co., Armonk, NY, USA).

## Results

Table 1 summarizes comparisons of the clinical findings between the KD with pyuria group (*n* = 48) and the UTI group (*n* = 118). In both groups, the proportion of boys was larger than that of girls (*P* = 0.085), and an older median age was observed in the KD with pyuria group than in the UTI group (*P* < 0.001). Of the total children, 67.8% (80/118) of the UTI group and 50.0% (24/48) of the KD with pyuria group were 2–24 months old (*P* = 0.032). The median duration of fever at diagnosis was longer in the KD with pyuria group than in the UTI group (*P* < 0.001). During hospitalization, 100.0% (118/118) of the UTI group and 66.7% (32/48) of the KD with pyuria group received intravenous antibiotics (*P* < 0.001).

**Table 1:** Comparison of clinical findings between the KD with pyuria group and the UTI group

	KD with pyuria (*n* = 48)	UTI (*n* = 118)	*P* value
Age, months	23.0 (9.0–33.8)	4.6 (3.0–8.1)	< 0.001
Male, *n* (%)	33 (68.8%)	64 (54.2%)	0.085
2–24 months of age, *n* (%)	24 (50.0%)	80 (67.8%)	0.032
Duration of fever at diagnosis, days	5.0 (4.0–5.0)	2.0 (1.0–2.0)	< 0.001
Length of hospital stay, days	6.0 (5.0–7.0)	5.0 (5.0–6.0)	0.105
Antibiotic usage, *n* (%)	32 (66.7%)	118 (100.0%)	< 0.001

Median (interquartile range; 25th–75th percentile); *KD* Kawasaki disease, *UTI* Urinary tract infection

Table 2 summarizes comparisons of the laboratory findings between the KD with pyuria group and the UTI group. In blood tests, both groups showed an increase in markers for acute inflammation, such as WBC count and CRP level, but the median value of CRP was higher in the KD with pyuria group than in the UTI group (*P* < 0.001). The median values for platelet count (*P* = 0.036) and albumin level (*P* = 0.003) were lower, but those of AST (*P* < 0.001) and ALT levels (*P* < 0.001) were higher in the KD with pyuria group than in the UTI group. More than 70.0% of the KD with pyuria group, but less than 20.0% of the UTI group possessed an elevated AST (> 40 IU/L) or ALT (> 40 IU/L) level (*P* < 0.001). On urinalysis, 40.7% (48/118) of the UTI group had a positive nitrite test, but 0.0% (0/48) of the KD with pyuria group had a positive nitrite test (*P* < 0.001). Hematuria was more common in the UTI group than in the KD with pyuria group (*P* = 0.001).

**Table 2:** Comparison of laboratory findings between the KD with pyuria group and the UTI group

	KD with pyuria (*n* = 48)	UTI (*n* = 118)	*P* value
Hemoglobin, g/dL	11.3 (10.6–12.2)	11.0 (10.3–11.6)	0.060
WBC count, × 10^9^ /L	14.7 (11.1–18.6)	14.4 (11.3–20.4)	0.753
Platelet count, ×10^9^/L	357 (292–426)	390 (323–466)	0.036
CRP, mg/L (normal < 5 mg/L)	94 (58–121)	45 (23–88)	< 0.001
Sodium, mmol/L	136 (135–137)	136 (135–138)	0.454
Albumin, g/L	39 (37–41)	41 (39–43)	0.003
AST, IU/L	52 (40–193)	35 (30–40)	< 0.001
ALT, IU/L	109 (30–258)	23 (17–32)	< 0.001
Elevated AST (> 40 IU/L), *n* (%)	34 (70.8%)	23 (19.5%)	< 0.001
Elevated ALT (> 40 IU/L), *n* (%)	34 (70.8%)	14 (11.9%)	< 0.001
Positive nitrite test, *n* (%)	0 (0.0%)	48 (40.7%)	< 0.001
Hematuria, *n* (%)	10 (20.8%)	59 (50.0%)	0.001
Proteinuria, *n* (%)	7 (14.6%)	29 (24.6%)	0.157

Median (interquartile range; 25th–75th percentile); *KD* Kawasaki disease, *UTI* urinary tract infection, *WBC* white blood cell, *CRP* c-reactive protein, *AST* aspartate transaminase, *ALT* alanine transaminase

Of the children with KD and pyuria, 11/48 (22.9%) cases of incomplete KD, 10/48 (20.8%) cases of unresponsiveness to IVIG therapy, and 8/48 (16.7%) cases of CALs (coronary artery dilatation or aneurysm) were identified.

## Discussion

KD, the leading cause of childhood acquired heart disease in developed countries [3, 4], and UTI, the most common serious bacterial infection in children [14, 15], are different disease entities and show distinctive clinical characteristics. However, KD is sometimes confused with UTI in actual practice because both can present with pyuria and CRP elevation [1, 14].

Wu et al. [10], reporting this diagnostic confusion, noted that pyuria and CRP elevation without concomitant signs suggestive of KD were the initial presenting signs, and children with KD were misdiagnosed as having UTI and received treatment with antibiotics. Similarly, in the present study, two-thirds of the KD with pyuria group received antibiotics, because the clinical and laboratory findings at the initial stage did not meet the diagnostic criteria for KD but suggested UTI. Considering the epidemiologic prevalence of UTI, febrile children with pyuria and CRP elevation require empiric antibiotics and proper work-up for UTI [14, 17]. However, when children suspected of having UTI show persistent fever despite proper antibiotic therapy, KD should be one of the differential diagnoses [12, 18]. Likewise, children with cervical lymphadenitis as the initial presentation can be misdiagnosed as having bacterial lymphadenitis [19, 20]. A high index of suspicion is particularly important in children initially presenting with pyuria or cervical lymphadenitis because delayed diagnosis is common in these clinical scenarios, and these children are at high risk of developing CALs [1, 19].

To minimize this diagnostic delay, we investigated the clinical and laboratory findings at the initial stage of disease that can differentiate KD from UTI in febrile children with pyuria and CRP elevation. Because the diseases have different age prevalence, age is considered the most important demographic characteristic [1, 14]. However, both KD and UTI would be included as diagnostic considerations in a certain age group. In the present study, one-half of the KD with pyuria group and two-thirds of the UTI group were 2–24 months old. Among the laboratory findings at the initial stage (i.e., on the day of admission), we found that elevated liver enzymes were more specific to KD than to UTI, but a positive nitrite test was more specific to UTI than to KD. Our findings can be used as diagnostic clues to differentiate KD from UTI. For example, if a febrile child with pyuria and CRP elevation shows both elevated liver enzymes and a negative nitrite test, there is a high possibility that the patient has KD. Conversely, if the same child shows both a normal range of liver enzymes and a positive nitrite test, it is likely that the patient has UTI.

Pyuria occurs not only in children with UTI, but also in those with KD [15, 21]. Pyuria in most children with KD is sterile and accompanied by a negative nitrite test [13, 21]. However, some children with KD can show bacterial pyuria and/or a positive nitrite test, indicating that there is a possibility of a coexisting UTI with KD in febrile children with pyuria and CRP elevation [17, 22]. The clinical significance of pyuria observed in KD is controversial, but the presence of pyuria might be associated with the severity of systemic inflammation or coronary complications [8–10]. Compared to nationwide data from the Republic of Korea [3], the KD with pyuria group in our study showed a relatively large percentage of cases that were both unresponsive to IVIG therapy (20.8% vs 10.8%, respectively) and developed CALs (16.7% vs 10.8%, respectively).

The present study has several limitations. First, the study was small-sized, retrospective, and conducted in a single center. Therefore, large-scale prospective studies are needed to support our findings. Second, UTI may have been over-diagnosed because most urine samples in our study were collected using a urine bag. Third, cases in which KD was accompanied by UTI may have been included in our study.

## Conclusions

We found that elevated liver enzymes are more specific to KD than to UTI, but a positive nitrite test is more specific to UTI than to KD. When a febrile child with pyuria and CRP elevation does not show the typical features of KD, our findings can be used as diagnostic clues to differentiate KD from UTI.

## Abbreviations

ALT: Alanine transaminase
AST: Aspartate transaminase
CRP: C-reactive protein
IVIG: Intravenous immunoglobulin
KD: Kawasaki disease
UTI: Urinary tract infection
WBC: White blood cell
